# Positional Differences in Peak- and Accumulated- Training Load Relative to Match Load in Elite Football

**DOI:** 10.3390/sports8010001

**Published:** 2019-12-23

**Authors:** Ivan Baptista, Dag Johansen, Pedro Figueiredo, António Rebelo, Svein A. Pettersen

**Affiliations:** 1 School of Sport Sciences, University of Tromsø—The Arctic University of Norway, 9037 Tromsø, Norway; svein.arne.pettersen@uit.no; 2 Computer Science Department, University of Tromsø—The Arctic University of Norway, 9037 Tromsø, Norway; dag.johansen@uit.no; 3 Portugal Football School, Portuguese Football Federation, 1495-433 Oeiras, Portugal; pedro.figueiredo@fpf.pt; 4 Research Center in Sports Sciences, Health Sciences and Human Development, CIDESD, University Institute of Maia, ISMAI, 4475-690 Maia, Portugal; 5 Center of Research, Education, Innovation and Intervention in Sport, Faculty of Sports, University of Porto, 4200-450 Porto, Portugal; anatal@fade.up.pt

**Keywords:** external load, accelerations, high-intensity runs, sprints, microcycle, playing position

## Abstract

Quantification of training and match load is an important method to personalize the training stimulus’ prescription to players according to their match demands. The present study used time-motion analysis and triaxial-accelerometer to quantify and compare: a) The most demanding passages of play in training sessions and matches (5-min peaks); b) and the accumulated load of typical microcycles and official matches, by playing position. Players performance data in 15 official home matches and 11 in-season microcycles were collected for analysis. Players were divided into four different playing positions: Centre-backs, wing-backs, centre midfielders, and centre forwards. The results show that match demands were overperformed for acceleration counts (acc_counts_) (131%–166%) and deceleration counts (dec_counts_) (108%–134%), by all positions. However, relative to match values, training values for sprint distance (sprint_dist_) and high-intensity run distance (HIR_dist_) were considerably lower (36%–61% and 57%–71%) than for accelerations and decelerations. The most pronounced difference on the 5-min peaks was observed in sprints (sprint_peak_), with wing-backs achieving during the microcycle only 64% of the sprint_peak_ in matches, while centre backs, centre midfielders, and centre forwards levelled and overperformed the match values (107%, 100%, and 107%, respectively). Differences observed across playing positions in matches and microcycles underline the lack of position specificity of common training drills/sessions adopted by coaches in elite football.

## 1. Introduction

Objective data and time-motion analysis are used by coaches and practitioners to characterize the physical demands of training sessions and matches, allowing training load (TL) and training specificity analysis. This data may provide valuable information when designing and optimizing training programs [1]. Nevertheless, even though general physical demands of match play are well known, there is a great variation across playing positions [2,3] and the position-specific load needs to be taken into consideration when designing and implementing training program cycles. Of particular importance is the potential ability that objective data provides for personalized prescription of TL in a cohort of players following the same overall training regime. 

Several studies have focused on the match load (ML) of professional football players [4,5,6,7]. However, in contrast to ML, information about the TL in elite players is scarce. Furthermore, important physical variables such as accelerations, decelerations, and peaks of high-intensity runs and sprints have been neglected in some previous research. Managing TL according to the average ML of the team is not sufficient, and new approaches are needed in order to fulfil the law of training specificity [8]. In fact, previous research [9,10] has concluded that athletes become underprepared for the most demanding phases of play (the most intense 5 min of a football match for a certain physical variable) if their training programs only focus in replicating the average demands of competition. The need for a deeper understanding of ML, as the analysis of peaks of intensity according to playing position, is then fundamental to better prepare the athletes for the physical demands of competition.

Only recently, some studies have analyzed the TL of professional football players and most of this research has paid special attention to the quantification of the TL in different microcycles [11,12] and to the comparison of unequal sessions within the same microcycle [13,14,15,16], using the “match-day minus or match-day plus” (MD-; MD+) approach [17]. However, the accumulated weekly TL relative to ML is still unclear since trainings and matches are usually measured with different tracking systems [16], which raises challenges regarding the validity of comparisons. Previous research [16] has attempted to perform comparisons between TL and ML using the same tracking system. Nevertheless, despite interesting results, only a few and non-official matches, with a different duration than official matches, were analyzed.

To the best of our knowledge, there are only two studies that have compared weekly training demands with match demands [12,16]. However, these studies have failed to consider the most demanding passages of play and the players playing positions while using official matches for comparison. Therefore, the aims of the present study were to quantify and compare: a) The most demanding passages of play in training sessions and matches; b) the accumulated load of typical training weeks (7-day microcycles) and official matches by playing position. We hypothesize that high-intensity runs (HIR) and sprint distances will present considerably lower training/match ratios when compared to accelerations and decelerations, and that the most physically demanding playing positions in the match will present lower training/match ratios than less demanding positions (mainly taking into considerations the overuse of small-sided games (SSG) and/or exercises played in relatively small areas in training sessions).

## 2. Materials and Methods

### 2.1. Subjects

With approval from the Norwegian Centre for Research Data and written informed consent from players, 18 male football players from the first team (highest level) of a Norwegian elite club took part in the study. Data from 15 official home matches and 11 in-season microcycles were collected for analysis. Players were divided into four different playing positions: Centre-backs (CB) (n = 4; match observations M_obs_ = 42; training observations T_obs_ = 141), wing-backs (WB) (n = 3; M_obs_ = 21; T_obs_ = 101), centre midfielders (CM) (n = 5; M_obs_ = 40; T_obs_ = 162), and centre forwards (CF) (n = 6; M_obs_ = 32; T_obs_ = 133). These positions were chosen according to the team’s tactical formation (1-3-5-2). All data was anonymized prior to the analyses.

### 2.2. Procedures

TL and ML data were collected using a stationary radio-based tracking system (ZXY Sport Tracking System, Trondheim, Norway)—specifications below. Match activity profiles, per position, in 15 official home matches during the 2018 season were characterized. Match data (excluding the warm-up) was analyzed only if: (a) Players completed at least 60 min of the match [16], and (b) the player played all the time in the same position. Match activity based on samples of less than 90 min were extrapolated to 90 min. We adapted the inclusive and extrapolation criteria from Stevens et al. [16], using the match data from players who played for at least 60 min. External load data of 11 typical microcycles (4 football training sessions within the 6-day period between matches) were collected and analyzed per position. Players without M_obs_ were not included in the sample, and T_obs_ from players who did not finish the training session were also excluded from the analysis. All training sessions were composed of warm-up exercises and a combination of technical drills, football conditioning games (SSG), finishing drills, and tactical exercises.

The team used in this study rarely played more than one match per week (participating only in the national league and cup). However, many breaks during the season (FIFA International Match Calendar, Summer break, etc.) led to a smaller number of “typical weeks” tracked (1 match per week with 6 full days between matches) [16,18] than what was expected. These typical microcycles often included 2 days-off (MD+1 and MD-2) and 4 training sessions. Only the main team sessions were considered. This refers to the training sessions where both starting and non-starting players trained together. Consequently, other types of sessions were excluded from analysis, including recovery sessions (MD+1), individual and conditioning training, as well as additional training for non-starters (MD+1). The matches and training sessions were all played on the same artificial grass surface (Alfheim Stadium, Tromsø, length = 110 m; width = 68 m).

### 2.3. Data Collection and Data Analysis

Each player used a belt, with an electronic sensor system, around their waist. Previous studies [19] have described the accuracy and reliability of the system in measuring player’s physical performance. The tags were activated or stopped when the trainings and matches started or finished from a remote control (laptop or tablet) and no manual procedure was needed.

Physical parameters analyzed included: Number of accelerations (acc_counts_), number of decelerations (dec_counts_), distance covered in HIR (HIR_dist_), distance covered in sprint (sprint_dist_), 5-min peak of accelerations (acc_peak_), 5-min peak of deceleration (dec_peak_), 5-min peak of HIR distance (HIR_peak_), and 5-min peak of sprint distance (sprint_peak_) [6,16,20,21]. Cumulative load per variable was calculated by summing the values of the 4 training sessions of each week/microcycle.

The “peak” variables refer to the values observed (frequency of accelerations or decelerations and meters for HIR or sprints) on the 5-min period of the match (individualized for each player) where such variables presented the highest values. The use of 5-min peak periods is in line with previous research [20,21,22,23,24], which used the same duration when analyzing the most intense periods of football matches. 

The speed thresholds for HIR (≥19.8 km h^−1^) and sprinting (≥25.2 km h^−1^) were chosen according to previous research [20,25,26,27].

### 2.4. Statistical Analysis

The lm4, lsmeans, and psychometric packages in the software [28] were used to conduct all the statistical analyses. Differences in local positioning measurement (LPM)-derived variables (sum or peak) between training and match by position were accessed through a linear mixed-effects model with restricted maximum likelihood estimations. The fixed effects in the models included session type, playing position, and interaction term, while “athlete ID” was included as a random effect. Thus, each athlete had a subject-specific intercept. An α-level of 0.05 was used as the level of significance for statistical comparisons. Unless otherwise stated, all the results are presented as mean and standard deviation. Moreover, the Tukey method was applied to adjust the multiple comparisons. The t statistics from the mixed models were converted to effect size correlations [29]. Effect sizes were interpreted as <0.1, trivial; 0.1–0.3, small; 0.3–0.5, moderate; 0.5–0.7, large; 0.7–0.9, very large; 0.9–0.99, almost perfect; 1.0, perfect [30].

Training data (cumulative load per variable and 5-min peaks of the whole microcycle) were also presented as a percentage of estimated match values (100%). To use the estimated match values, the average of each variable across the 15 matches tracked was calculated and then considered as 100%.

## 3. Results

### 3.1. Accumulated Training Load

Table 1 presents the comparison between the match and microcycle values for different variables across playing positions. CF performed significant more accelerations and decelerations during training sessions (112.3 ± 5.8 and 94.1 ± 5.9) than in matches (78.5 ± 6.2 and 74.3 ± 6.3, respectively). Furthermore, the inverse was observed in HIR_dist_ and sprint_dist_, with CF covering higher distances during matches (897.1 ± 62.6 and 171.7 ± 1.0 m) compared to trainings (561.0 ± 59.3 and 104.6 ± 0.9 m, respectively).

During the microcycles, CB accumulated significantly higher acc_counts_ (89.0 ± 6.0) and dec_counts_ (73.6 ± 6.4) than in matches (61.1 ± 6.0 and 55.1 ± 6.4, respectively). However, the opposite was observed regarding HIR_dist_ and sprint_dist_ performed in matches (479.5 ± 65.9 and 86.3 ± 1.0 m) being considerably higher than in microcycles (340.7 ± 65.8; 42.6 ± 1.0 m, respectively).

Even though WB did not present significant differences in acc_counts_ neither in dec_counts_, statistically lower values of HIR_dist_ and sprint_dist_ were observed in the microcycles (564.9 ± 76.4 and 85.8 ± 1.2 m) than in matches (984.7 ± 82.9 and 238.2 ± 1.3 m, respectively). Moreover, CM presented statistical differences between matches and microcycles only in acc_counts_ (54.2 ± 6.0 and 90.2 ± 5.5) and HIR_dist_ (615.4 ± 63.4 and 374.1 ± 59.9 m, respectively). Despite not all differences being statistically significant, a clear pattern is possible to identify in Table 1, with all playing positions presenting lower acc_counts_ and dec_counts_ in matches than in microcycles and higher HIR_dist_ and sprint_dist_ in matches than in microcycles.

**Figure 1:**
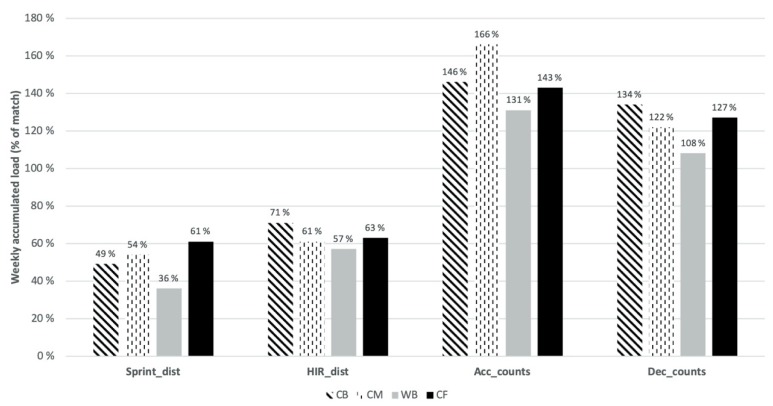
Microcycle accumulated load in percentage of match load.

Figure 1 shows the estimated cumulative load per variable during a microcycle expressed as a percentage of tracked match values (100%). The match demands were largely overperformed for acc_counts_ (131%–166%) and dec_counts_ (108%–134%), by all the playing positions. However, relative to match values, training values for sprint_dist_ and HIR_dist_ were considerably lower (36%–61% and 57%–71%) than those previously reported for accelerations and decelerations.

### 3.2. Most Demanding Passages of Play (5-min Peaks)

Significant differences between matches and trainings were observed only in acc_peak_ for CB (6.4 ± 0.4 and 7.5 ± 0.4) and CM (6.2 ± 0.4 and 7.7 ± 0.4, respectively) (Table 2). However, WB presented slightly higher values of HIR_peak_ and sprint_peak_ in matches (119.0 ± 9.6 and 56.7 ± 6.7 m) than in trainings (84.3 ± 8.6 and 36.3 ± 6.0 m, respectively). All the other playing positions and peak variables presented similar values between matches and microcycles.

**Figure 2:**
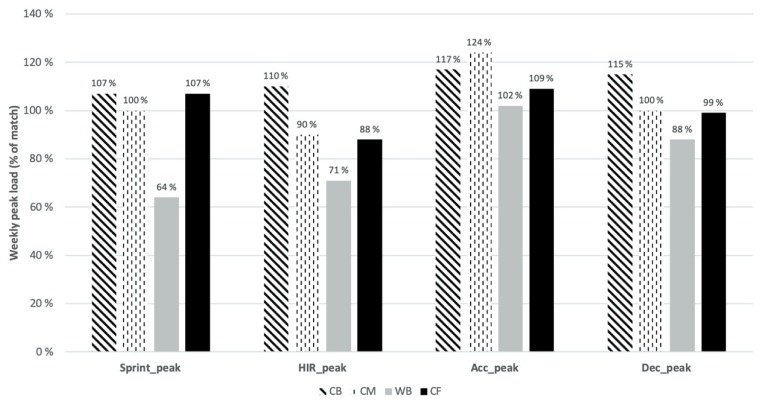
Microcycle 5-min peaks in percentage of match peaks.

Moreover, Figure 2 shows the estimated training 5-min peaks of the whole microcycle, expressed as a percentage of estimated match values (100%). For acc_peak_ and dec_peak_, the percentages did not differ largely between playing positions (range: 102%–124% and 88%–115%, respectively), with CB and CM performing at slightly higher values (relative to their specific match demands) than WB and CF. However, the biggest difference observed between playing positions is for sprint_peak_, with WB achieving, during the microcycles, only 64% of the most demanding 5-min sprint distance in matches, while CB, CM, and CF levelled and overperformed the match values (107%, 100%, and 107%, respectively).

## 4. Discussion

In the present study, we objectively quantified and compared, per playing position, the weekly training load and most demanding passages of play (5-min peaks) with match demands. Consistent with our hypothesis, the number of accelerations and decelerations during training weeks were considerably higher than the match values, while the distances ran at the most demanding speed thresholds (HIR and sprints) were much lower in microcycles than in matches. In general, the results reveal a lack of consistency between positions in the accumulated training load and in the most demanding 5-min peaks, relative to their specific match demands. Table 1 and Table 2 reveal that while the training demands were statistically different from the match demands for some positions (e.g., CB and CF in dec_counts_), the same was not observed for other positions, where the differences between training and matches were insignificant (e.g., CM and WB in dec_counts_). According to previous research [16,18], the interpretation of training load data is facilitated when match load is used as a reference, helping the training prescription as well as the communication between coaches and players. In Figure 1 and Figure 2 we used the cumulative load and the 5-min peaks during a microcycle, expressed as a percentage of estimated match values (100%).

Figure 1 clearly shows that match demands were overperformed for acc_counts_ (131%–166%) and dec_counts_ (108%–134%) but underperformed for HIR_dist_ (57%–71%) and sprint_dist_ (36%–61%). These results are somewhat in line with previous studies with Dutch [16] and Portuguese [18] football teams, where similar discrepancies between the accumulated weekly load of different variables were reported. These findings suggest that nowadays, the training drills used tend to emphasize some physical variables, such as accelerations and decelerations, and neglect others, like HIR_dist_ and sprint_dist_. Ade et al. [31] found that SSG and exercises played in small areas increased the number of accelerations and decelerations, when compared with running-based drills, but the latter requested more HIR and sprints. Gabbett et al. [32] also suggested that, since SSG do not simulate high-intensity and sprint demands of official matches, such exercises should be complemented with game-specific drills where the high-intensity and sprint demands of international competitions are represented.

Even though the ability to perform high-intensity exercise has been proven to be strongly correlated with success in football [21,29], some research in different collective sports, including football [33,34,35,36], defend that the concept of “train as you play” is highly impractical, due to the high match demands and the associated injury risk. Indeed, differences between microcycles and matches should be expected, given that simply reproducing match demands in trainings would oversimplify the complex process of developing elite players [34,37]. However, it is very unlikely that trainings with consistently lower distance covered in the most demanding speed thresholds, compared with competition, offer an optimal stimulus for players adaptation to the match demands [34]. Moreover, the argument of risk of overtraining, used to not raise the frequency and distance run at high-speed thresholds during trainings, may be rebutted with the higher metabolic demands as well as the greater neural activation of the working muscles when performing accelerations, decelerations, and changes of direction, compared to constant speed running [15,17,38,39].

In a study of a Spanish football team [10], the authors concluded that the physical demands of the most demanding passages of play are position-dependent. Therefore, developing training programs based on absolute or average match values only may limit specificity and underestimate the real demands of the most demanding passages of competition. Figure 2 shows that when taken into consideration only the most demanding 5 min, differences between variables were minimized and match values were replicated in trainings. However, one exception can be spotted, with WB performing considerably lower values of sprint_peak_ (64%) and HIR_peak_ (71%) than the other positions, suggesting that the players in this playing position may not be prepared for the worst-case scenario in matches. The fact that WB were required to perform longer distances of HIR_peak_ (119.0 ± 9.6) and sprint_peak_ (56.7 ± 6.7) in matches than all the other positions means that training stimulus for this playing position should be increased if coaches and practitioners aim to prepare these players for extreme events that occur in matches. A higher level of training specificity is needed in order to meet the match demands of all playing-positions. Such specificity can be achieved through on-field training methods that aim to match or exceed the demands of competition in all the performance components (physical, tactical, technical, and psychological) [40]. 

Despite the novelty and practical implications for football practitioners given by this study, some limitations must be considered. The common limitation within the literature, when studying professional and elite players (small sample size), was one of the challenges faced in this research, as well as the fact that only one team was analyzed. This means that true differences might be masked due to a statistical type 2 error, and the coach’s training philosophy may also have contributed for differences observed. Another limitation relates to one of the difficulties when using applied research, which was the fact that only 11 microcycles were tracked, since the team’s match schedule and coach’s decisions about the structure of the microcycle (microcycles with less or more than four training sessions) could not be controlled by the researchers. Nevertheless, this choice was made to ensure that precise values of the most common types of microcycles in elite football (four training sessions) [12,13,16,35,41,42] were obtained. Moreover, any internal load measures (e.g., heart-rate, rated perceived exertion, etc.) were considered since it was out of the scope of this study. Finally, more specific comparisons with the results of previous research is difficult to conduct, since currently there is little consensus regarding the acceleration and deceleration thresholds used in team sports [43] and because of the different tracking systems used.

Future research should also attempt to better contextualize training loads, so practitioners can visualize the specific physical demands of different exercises. Nevertheless, the findings presented in this study provide important and novel information which may be used by practitioners to adapt their strategies to the need of a more position-specific training methodology.

## 5. Conclusions

Differences observed across playing positions in matches and microcycles underline the lack of position specificity of common training drills/sessions adopted by coaches in elite football. Position-specific training is likely to appear if the players typically train in the same positions in which they will compete [40], and to do so, we recommend: a) The use of bigger SSG (e.g., >6 vs. 6) in practices, since larger playing areas will influence the distances that can be covered at high speeds; and b) to complement these sessions with running-based drills. It is important to emphasize that there are many possible ways to achieve this type of specificity and we have only provided a few suggestions.

Coaches and practitioners must keep in mind that the absolute TL accumulated by players of different positions should not be a tool for measurement. Alternatively, analyzing the relative TL (according to the match demands) may be a much better and valuable way of managing and evaluating the players periodization. For instance, applying similar sprint_dist_ in trainings to all the players regardless their playing positions would most likely lead to underloading WB and CF (most physically demanding positions) and overloading CB and CM (less physically demanding positions). Such differences are likely to affect performances and increase the injury risk. Furthermore, differences observed across playing positions in matches and microcycles underline the importance of the individualization of the physical training, within the collective periodization. Therefore, further research is needed to represent a broader overview of the relationship between TL and ML in professional football as well as the effects of different periodization strategies.

## Figures and Tables

**Table 1:** Mean and ± SD values of physical variables, per playing position, according to the type of sessions (matches and microcycles).

Variables		Acc_counts_	Dec_counts_	HIR_dist_	Sprint_dist_
CB	Match	61.1 ± 6.0	55.1 ± 6.4	479.5 ± 65.9	86.3 ± 1.0
	Microcycle	89.0 ± 6.0 *^#^	73.6 ± 6.4 *^#^	340.7 ± 65.8 ^#^	42.6 ± 1.0 *^#^
CM	Match	54.2 ± 6.0	60.6 ± 6.3	615.4 ± 63.4	79.4 ± 1.0
	Microcycle	90.2 ± 5.5 *^$^	73.8 ± 5.9 ^#^	374.1 ± 59.9 *^#^	42.8 ± 0.9 ^#^
WB	Match	79.8 ± 8.0	82.3 ± 8.2	984.7 ± 82.9	238.2 ± 1.3
	Microcycle	104.3 ± 7.0 ^#^	89.1 ± 7.5 ^#^	564.9 ± 76.4 *^$^	85.8 ± 1.2 *^$^
CF	Match	78.5 ± 6.2	74.3 ± 6.3	897.1 ± 62.6	171.7 ± 1.0
	Microcycle	112.3 ± 5.8 *^#^	94.1 ± 5.9 *^#^	561.0 ± 59.3 *^$^	104.6 ± 0.9 *^#^

HIR_dist_ and sprint_dist_ values presented in meters. * Statistically significant difference between match and microcycle (*p*-value < 0.05); # Small effect size (0.1–0.3); $ Moderate effect size (0.3–0.5).

**Table 2:** Mean and ± SD values of 5-min peaks in different physical variables, per playing position, according to the type of sessions (matches and microcycles).

Variables	CB		CM		WB		CF	
	Match	Microcycle	Match	Microcycle	Match	Microcycle	Match	Microcycle
Acc_peak_	6.4 ± 0.4	7.5 ± 0.4 *^#^	6.2 ± 0.4	7.7 ± 0.4 *^#^	8.4 ± 0.5	8.6 ± 0.5	8.0 ± 0.4	8.7 ± 0.4 ^#^
Dec_peak_	6.2 ± 0.3	7.1 ± 0.3 ^#^	6.6 ± 0.3	6.6 ± 0.3	8.6 ± 0.5	7.6 ± 0.4 ^#^	7.6 ± 0.4	7.5 ± 0.3
HIR_peak_	74.7 ± 6.9	82.0 ± 7.1	86.3 ± 7.0	77.4 ± 6.9	119.0 ± 9.6	84.3 ± 8.6 ^#^	104.6 ± 7.8	91.9 ± 7.4
Sprint_peak_	32.9 ± 4.9	35.1 ± 5.0	32.3 ± 5.0	32.2 ± 4.8	56.7 ± 6.7	36.3 ± 6.0 ^#^	40.3 ± 5.3	43.3 ± 5.0

acc_peak_ and dec_peak_ values presented in frequency (counts). HIR_peak_ and sprint_peak_ values presented in meters. * Statistically significant difference between match and microcycle (*p*-value < 0.05); # Small effect size (0.1–0.3).
